# Treatment of recurrent facial ulcerations and dysesthesias from trigeminal trophic syndrome with chronic transcutaneous vagus nerve stimulation

**DOI:** 10.1016/j.jdcr.2026.03.035

**Published:** 2026-03-23

**Authors:** William J. Nahm, Vincent Falanga, Ryan Chen, Goranit Sakunchotpanit, Ki-Joong Kim, David A. Lee, John Koo

**Affiliations:** a New York University Grossman School of Medicine, New York, New York; b Department of Dermatology, Boston University Chobanian and Avedisian School of Medicine, Boston, Massachusetts; c Department of Biochemistry & Cell Biology, Boston University Chobanian and Avedisian School of Medicine, Boston, Massachusetts; d University of Massachusetts Chan Medical School, Worcester, Massachusetts; e Tufts University School of Medicine, Boston, Massachusetts; f Department of Korean Medicine, Institute of Bioscience and Integrative Medicine, Daejeon University, Daejeon, South Korea; g Damascus Dermatology & Skin Surgery Center, Damascus, Maryland; h Department of Dermatology, University of California, San Francisco, California

**Keywords:** dysesthesia, gabapentin, N-acetyl cysteine, naltrexone, sertraline, transcutaneous, trigeminal trophic syndrome, ulceration, V2 dermatome, vagus nerve stimulation

## Introduction

Trigeminal trophic syndrome (TTS) represents an uncommon sequela of trigeminal nerve damage, clinically defined by a triad of chronic facial ulceration, sensory loss, and abnormal sensations, distributed within the anatomical territory of the affected trigeminal nerve dermatome.^1^ The syndrome typically manifests following direct trauma to the trigeminal nerve or its central connections. However, other etiologies, including surgical intervention, cerebrovascular injury, neoplastic processes, and inflammatory conditions, have been documented.^2^ The pathognomonic facial ulceration develops secondary to repetitive self-manipulation of the V2 facial region, a behavior thought to arise from the combination of sensory deficit and dysesthetic symptoms. Ulceration typically affects the nasal ala but may involve other areas innervated by the trigeminal nerve. The nasal tip remains typically spared due to innervation by the medial anterior ethmoidal nerve rather than the trigeminal branches. This self-inflicted trauma perpetuates a cycle of tissue damage and impaired healing, often resulting in deep, crateriform lesions with rolled borders.^1^^,^^2^

Recently, vagus nerve stimulation (VNS) has demonstrated anti-inflammatory effects across various organs and shows therapeutic potential for inflammatory skin conditions,^3^^,^^4^ and has been found to inhibit trigeminal nociception in rodent models of episodic migraine, suggesting possible relevance to trigeminal dysesthesia symptoms.^5^

## Case report

**Fig 1:**
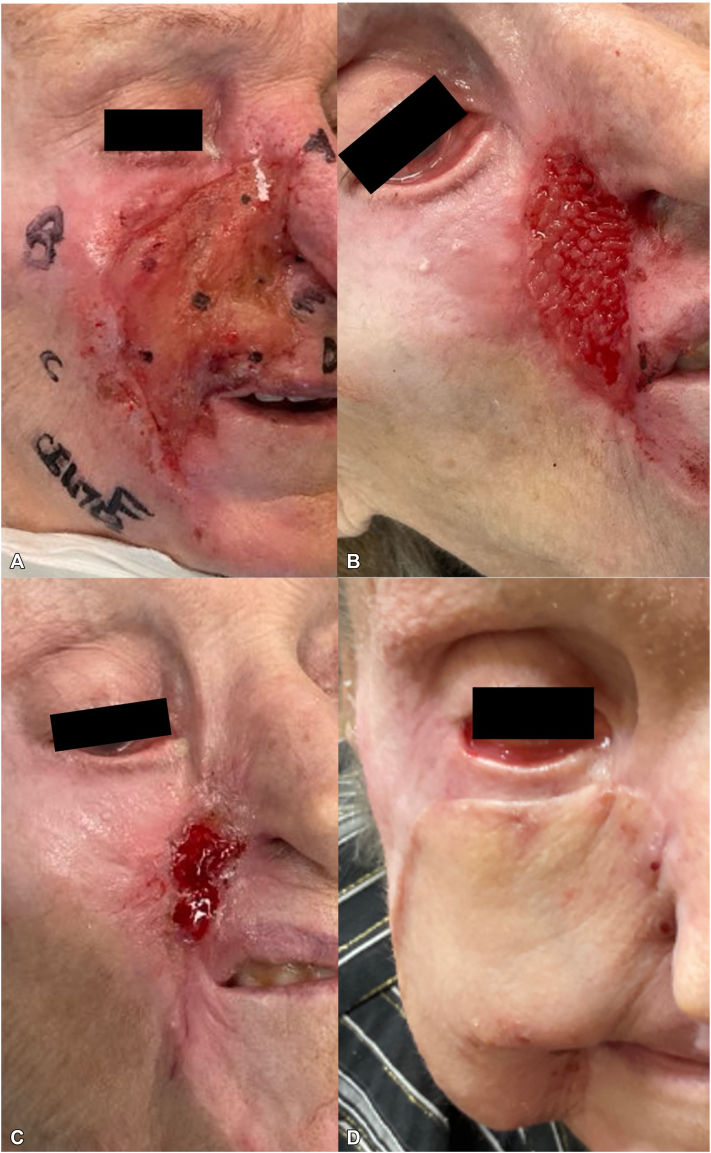
Initial presentation of trigeminal trophic syndrome (TTS) and treatment of patient. **A,** Large, angulated ulceration on the right medial cheek with right nasal erosion. The patient also had tarsorrhaphy of the right eye. **B,** After 3 weeks of neuromodulatory medications and placements of a human skin allograft, dramatic closure of the ulceration with wound contracture is observed. **C,** Almost complete closure of ulceration occurring after 1 month of treatment, with further wound contracture causing right lower eyelid ectropion and right upper lip superior displacement. **D,** Postoperative picture of the right cheek with a right submental pedicled flap from the neck, 3 months and 3 weeks after starting medication treatments. There are no dysesthesias and no ulceration.

We present a resistant case of TTS, where an 85-year-old female had a significant, angulated ulceration encompassing the medial aspect of the right cheek, upper lip area, and lateral aspect of the mouth, measuring 10.2 cm × 5.4 cm (Fig 1, *A*). The right nasal ala was completely eroded, revealing the medial aspect of the nostril (Fig 1, *A*). Moreover, she experienced right eye corneal ulcerations from extensive ectropion.

Nine years ago, she had a right-sided stroke, which left her with trigeminal numbness in the V2 distribution. Seven years poststroke, she had ulcerations in the V2 area from persistent scratching, progressing to what is seen presently. The patient’s primary complaint was the feeling of a “runny nose” on the right side, causing a constant urge to wipe the right side of the face and nose. Due to her ectropion and corneal ulceration from wound healing contraction, she had a tarsorrhaphy of the right eye with oculoplastic surgery. After 5 punch biopsies throughout the ulceration and ruling out other etiologies, we employed a daily course of naltrexone 50 mg, sertraline 25 mg, gabapentin 300 mg, N-acetyl cysteine 1000 mg, and every other week applications of a human skin allograft. The patient experienced a gradual loss of dysesthesia and improved wound closure within the first 2 weeks, with complete dysesthesia resolution and near-complete closure by 1 month (Fig 1, *B* and C).

The resultant V2 ulceration closure led to marked orofacial contracture, further ectropion of the right eyelid, and dramatic upward displacement of the superior lip, resulting in the inability to close the mouth completely on the right side (Fig 1, *C*).

Approximately 3 months and 3 weeks after initiating medications, plastic surgery was involved to release right cheek tissue contraction with a large right-sided submental pedicled flap from the neck (Fig 1, *D*), and several months later, oculoplastics placed a graft on the right lower eyelid area to repair ectropion lid insufficiency.

**Fig 2:**
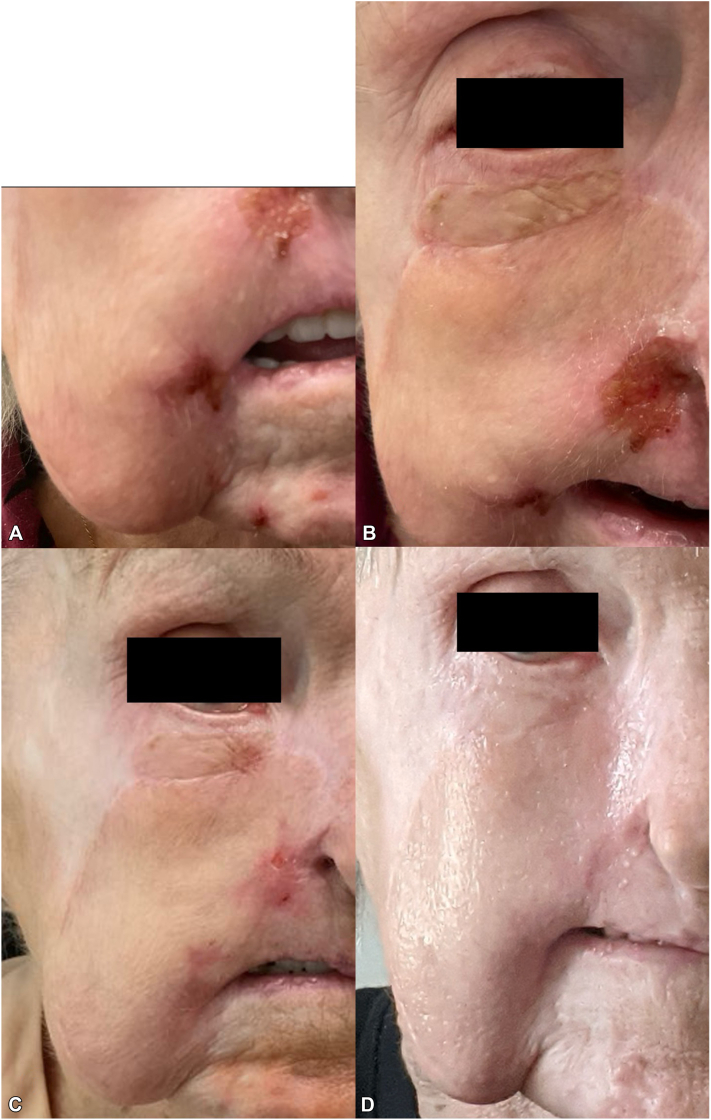
Recurrent ulcerations from trigeminal trophic syndrome (TTS) and introduction of vagus nerve stimulation (VNS). **A,** Return of ulceration and dysesthesia on the right nasal groove area, right side of mouth, and right chin after 7 months of neuromodulatory medication treatment **B,** Close-up of crusted ulceration on the right nasal ala and groove area after 7 months. Also, the outline of the right lower eyelid graft and the right cheek pedicle flap are seen. **C,** Complete resolution of ulcerations and dysesthesias with adjunctive treatment of contralateral cervical transcutaneous VNS for 1 month to 8 months after initial presentation. **D,** Continued lack of ulceration and dysesthesia after stopping neuromodulatory medications and implementing daily VNS for 6 months (14 months after initial presentation).

The patient did well with no dysesthesias or ulceration formation, but after 7 months, she started feeling sensations of a “runny nose” dysesthesia, lead to the development of superficial self-inflicted ulcerations in the right nasal groove area, lateral mouth area, and side of the chin (Fig 2, *A* and *B*).

The patient was offered referral to psychiatry for behavioral modification, but was unable to obtain treatment due to insurance issues. Due to reports of trigeminal neuralgia responding to VNS,^6^ the patient was offered and employed cervical (left-sided, contralateral) transcutaneous VNS (frequency 30 Hz, pulse width 300 μs) for 20 minutes per day at home using a handheld device. Gradual improvement in dysesthesia was noted over the first month of daily use, with complete resolution of dysesthesia and ulcerations observed at 1 month (Fig 2, *C*). No adverse effects from VNS were reported.

After 3 months, the patient discontinued the course of naltrexone, gabapentin, sertraline, and N-acetyl cysteine but continued employing VNS. The patient remained free of dysesthesia and ulceration for 6 months, after stopping all neuromodulatory medications (14 months from time of initial presentation), while still employing daily VNS (Fig 2, *D*).

Concurrently, the patient, while performing daily VNS, demonstrated improvement across multiple depressive symptom domains, including enhanced affect, restoration of capacity for pleasure, diminished irritability, and increased social engagement.

## Discussion

The resolution of dysesthesia and absence of ulceration within the V2 dermatome with VNS, both during neuromodulatory medication use and after their discontinuation, suggests that VNS may be an effective treatment for TTS. VNS has been shown to reduce glutamate levels in the trigeminal nucleus caudalis, and functional imaging and animal studies demonstrate that VNS suppresses spontaneous and evoked firing of trigeminocervical neurons, attenuates trigeminal autonomic reflexes, and modulates pain thresholds in trigeminal dermatomes, likely via central top-down pathways involving the hypothalamus, pontine nuclei, and spinal trigeminal nucleus.^6^, ^7^, ^8^ The VNS was performed on the contralateral side in our case because there have been reports of VNS triggering trigeminal neuralgia-like pain when performed ipsilaterally.^9^

The improvement in depressive symptoms correlates well with the observation that VNS attenuates inflammatory responses both peripherally (through alterations in cytokine levels) and centrally (by reducing microglial activation), a process known as the parasympathetic anti-inflammatory pathway. VNS inhibits TNF-α synthesis and reduces peak serum TNF-α levels in animal models of endotoxemia, and reduces central levels of inflammatory cytokines IL-1β, IL-6, and TNF-α while preventing hippocampal microglial activation.^10^ VNS also shows a 42% to 53.1% 2-year remission rate for refractory depression, with the antidepressant effects potentially mediated through restoration of inflammatory homeostasis.^10^

Given VNS's demonstrated capacity to suppress proinflammatory cytokines, its therapeutic applications may extend to other inflammatory dermatoses, such as psoriasis and atopic dermatitis, in which neuroimmune dysregulation plays a central pathophysiological role.

Transcutaneous VNS is a noninvasive, patient-administered modality that can be performed independently at home with minimal technical knowledge, offering a practical adjunctive option for TTS when conventional neuromodulatory pharmacotherapy is insufficient. The patient's most recent follow-up at 14 months from initial presentation demonstrated a positive clinical response, with sustained absence of dysesthesias and ulceration, and the treatment was well tolerated.

## Conflicts of interest

None disclosed.
